# Atlanta Medical College

**Published:** 1858-04

**Authors:** 


### EDITORIAL AND MISCELLANEOUS.

### Atlanta Medical College.

As at this season, the prospect for a class in this Institution is a matter of interest to many who receive this Journal, we would state that—notwithstanding the recent wide-spread financial embarrassment, from which indeed the country has not entirely recovered—there is scarcely a doubt that there will be a decided increase in the number of students at the next session. Although industrious efforts have been made in some quarters, since the last session, to discredit the Institution on account of the period of the year select[illegible] for the lectures, we can confidently state that the College has acquired more reputation and influence in its behalf in the last six months, than in any previous *year*.

We shall not be restrained by false delicacy, from stating that the medical men of the country have not allowed themselves to be deceived by the disingenuous effort which has been made to produce the impression, that to graduate a student in the *summer*, was necessarily to shorten the period of his study.

The effort upon the part of some to show that they had a higher rega[illegible] for the honor of the profession, than others, has been effectually met by the fact, that they had already brought the standard of requisitions for graduation down to its lowest point, and though making an effort to hold others to a strict adherence to a certain regulation, which can in no way secure qualification, it was notorious, that they recognized no obligation upon themselves—that, indeed, to attend two *winter* courses of lectures was the sum and substance of *their* standard.

Though we do not believe that *time* will ever make it certain, that unqualified men will not be allowed to graduate, yet, we are in favor of any period of study which the Colleges of the country will bind themselves to adopt and conform to, as an indispensable requisition, before a student shall be allowed to graduate.

We would, however, suggest, that the hope of elevating the standard of qualification lies in some other direction, and we feel strongly inclined to advocate—what we have intimated upon a former occasion as our disposition—a separation of the teaching and licensing power.

But, to take a practical and common sense view of this subject—to look at things just as they are—we know the fact that men will go into the medical profession, without regard to colleges, and the people will employ them with equally little regard to their opportunities or qualifications; under such circumstances it is the heighth of folly in our judgment, to expect either medical associations or colleges to *control* the proficiency of the *Doctors* of the land. If a board of medical men who were known to be qualified, could be appointed in each State, before whom, each applicant for license to practice in the State, was compelled by legislative enactment, to go for examination and permission to practice the profession, and in addition to this, the State Medical Societies, and the American Medical Association would require certain *attainments* as a requisition for membership in those bodies, (a part of which has been suggested by the Editor of the New Jersey Medical and Surgical Reporter,) under certain restrictions, something in the way of elevation of the profession might be accomplished. In the freedom prevailing in reference to entrance into any calling in the United States, there must be some higher inducement for men to make attainments in medical science, than the mere license or diploma, which gives permission to pursue the profession.

Notwithstanding these views, we consider it our duty to make every effort to send out properly qualified medical men, and to refuse to graduate those who are known to be incompetent: this has been our practice, and shall continue to be; and we can assure those who are pursuing the profession, that they can never receive the diploma of this College with a lower grade of qualifications than those required by the most respectable medical institutions in the country; nor, indeed, without a higher grade of qualification than that which we know to be sufficient in some *winter* schools.

